# Dynamics of mitochondrial membranes under photo-oxidative stress with high spatiotemporal resolution

**DOI:** 10.3389/fcell.2023.1307502

**Published:** 2023-11-17

**Authors:** Vincent Loriette, Alexandra Fragola, Sergei G. Kruglik, Susmita Sridhar, Antoine Hubert, François Orieux, Eduardo Sepulveda, Franck Sureau, Stephanie Bonneau

**Affiliations:** ^1^ ESPCI, PSL Research University, Sorbonne Université, CNRS, Laboratoire de Physique et D’Étude des Matériaux (LPEM), Paris, France; ^2^ Sorbonne Université, CNRS, Laboratoire Jean Perrin (LJP), Paris, France; ^3^ Centrale Supelec, Université Paris Saclay, CNRS, Laboratoire des Signaux et Systémes (L2S), Gif-sur-Yvette, France; ^4^ Sorbonne Université, Université Paris Cité, CNRS, Laboratoire de physique nucléaire et de hautes énergies (LPNHE), Paris, France

**Keywords:** structured illumination microscopy (SIM), live cell imaging, shape changes, dynamics, mitochondria, sub-organelles structures, oxidation, Chlorin-e6

## Abstract

In our study, we harnessed an original Enhanced Speed Structured Illumination Microscopy (Fast-SIM) imaging setup to explore the dynamics of mitochondrial and inner membrane ultrastructure under specific photo-oxidation stress induced by Chlorin-e6 and light irradiation. Notably, our Fast-SIM system allowed us to observe and quantify a distinct remodeling and shortening of the mitochondrial structure after 60–80 s of irradiation. These changes were accompanied by fusion events of adjacent inner membrane cristae and global swelling of the organelle. Preceding these alterations, a larger sequence was characterized by heightened dynamics within the mitochondrial network, featuring events such as mitochondrial fission, rapid formation of tubular prolongations, and fluctuations in cristae structure. Our findings provide compelling evidence that, among enhanced-resolution microscopy techniques, Fast-SIM emerges as the most suitable approach for non-invasive dynamic studies of mitochondrial structure in living cells. For the first time, this approach allows quantitative and qualitative characterization of successive steps in the photo-induced oxidation process with sufficient spatial and temporal resolution.

## 1 Introduction

Mitochondria are traditionally characterized as highly dynamic double-membrane organelles, exhibiting a wide range of shapes and morphologies, spanning from small spheres to intricate tubular networks with diameters typically measuring between 0.5 and 1 µm and lengths ranging from 1 to tens of micrometers. These organelles possess unique ultrastructural features, notably an inner membrane folding structure known as cristae, where the essential oxidative phosphorylation proteins are localized. These features support the coordination of respiratory complexes to generate cellular energy (Mannella, 2006; Owen et al., 2010). This multi-scale description of mitochondria underlies many significant variations in all these characteristics - size, number, dynamics, shape.—not only between different cell types but also within the same cell type, depending on the level of cellular activity. Even within the same cell, mitochondria can exhibit variations in morphology and activity. Moreover, recent studies have evidence that cristae possess a higher membrane potential compared to the surrounding boundary membranes, involving the presence of confined proton loops and distinct individual functionality of each crista within the same mitochondrion (Wolf et al., 2019). This arrangement entails distinctive physical, mechanical, and chemical hallmarks, such as an enrichment in cardiolipin of the membrane, which influence and support the specific architecture of the membrane surface (Patil et al., 2020). These characteristics have strong consequences at the microscopic level and lead to particular modifications in the diffusion processes of molecules, including protons and protein complexes, imposing, for example, in-plane or three-dimensional confinement effects. Specific alterations in mitochondrial cristae shape and network have also been linked to mitochondrial dysfunctions and are observed in various pathological and cellular aging scenarios (Vafai and Mootha, 2012; Siegmund et al., 2018). These findings put the focus on the intricate relationship between cristae shape and energy production, shedding light on the physiological significance of mitochondrial morphology, shape changes and dynamics.

Mitochondria play a pivotal role in balancing cellular energy metabolism, oxidative stress responses, and of the regulation of programmed cell death. Imbalances in redox processes can induce changes in membrane permeability, affecting the mitochondrial membrane potential and leading to the release of proapoptotic proteins from the mitochondrial intermembrane space. The internal organization of mitochondria emerges as a critical factor for the accurate functioning of this organelle. In the present study, our objective is to elucidate the dynamics of the intracellular mitochondrial membrane under specific oxidative stress induced by photoactivation in the presence of Chlorin-e6 (Ce6), a photosensitizer molecule. Ce6 was chosen due to its amphiphilic nature and high affinity for biological membranes. Its photo-induced oxidative efficiency has been extensively investigated within both lipid vesicles and cell cultures (Mojzisova et al., 2007; 2009; Heuvingh and Bonneau, 2009; Kerdous et al., 2011; Bour et al., 2019) and in clinical studies. Notably, Ce6 presents an absorption peak around 405 nm, allowing for superficial impact, and efficient antimicrobial therapy. As a second-generation photosensitizer, it ranks among the most widely utilized photosensitizers in photodynamic therapy (PDT), characterized by high efficacy and minimal dark toxicity. It has received approval for clinical trials in the PDT treatment of cancer and bacterial infections (Shakhova et al., 2018).

In pursuit of a comprehensive understanding, advanced microscopy techniques with enhanced resolution become the method of choice for examining the organization of the inner mitochondrial membrane within living cells during oxidative processes (Godin et al., 2014; Große et al., 2016; Salvador-Gallego et al., 2016; Segawa et al., 2020; 2020; Taguchi et al., 2021). Beyond spatial heterogeneity, the study also demands an examination of temporal variations in cell membranes. Stimulated Emission Depletion (STED) nanoscopy emerges as an appealing choice, offering high spatial resolution (∼50 nm) and a temporal resolution of approximately 1 frame per second (fps). Nevertheless, achieving this enhanced resolution technique necessitates high-intensity illumination, as the fluorescence within the donut-shaped area has to be converted into stimulated emission through powerful laser excitation (Stephan et al., 2019). Consequently, this approach often results in rapid photobleaching of common mitochondrial fluorescence markers like Mitotracker Green™. To address this limitation, superphotostable markers such as MitoPB Yellow and mitochondrial enhanced squaraine variant probes have been introduced, enabling STED spatiotemporal observations of mitochondrial dynamics in HeLa cells at 0.77 fps with a laser power of 100 mW (Wang et al., 2019) and 0.3 fps with a laser power of 8.96 mW (Yang et al., 2020). Nevertheless, these cutting-edge super-resolution approaches still present challenges when used on living cells, due to the biological effects of high irradiation (more than 3 109 W/cm2), including characteristic mitochondrial swelling, cristae loss, and membrane ruptures (Wang et al., 2019), as well as rapid changes in mitochondrial width in control cells (Yang et al., 2020). Consequently, these methods are not fully suitable for studying mitochondrial dynamics and shape changes in living cells, especially for examining its modifications during photo-induced oxidation compared to control cells. Considering these challenges and limitations, among the array of advanced enhanced-resolution imaging methods, low-excitation SIM microscopy emerges as the most promising approach, providing the capability for quantitative analysis of spatiotemporal mitochondrial dynamics under various controlled cellular conditions, with low excitation-related biological effects. A recent study has demonstrated the feasibility of long-term mitochondrial dynamics imaging using SIM (Liu et al., 2021). In order to adapt this method to the temporal constraints imposed by our experiments, we have developed a custom Fast-SIM setup, innovatively enhancing the acquisition rate and enabling rapid, large field-of-view (FOV) imaging with minimal sample irradiation (0.3 W/cm2). The Fast-SIM configuration we present can capture up to 2 frames per second, achieving a lateral resolution of 130 nm within an 85 μm × 85 µm FOV (Nadova et al., 2016; Orieux et al., 2017).

This experimental approach allows us to gather both qualitative and quantitative data for a most comprehensive characterization of mitochondrial membrane remodeling processes associated with photoinduced oxidative stress. Our quantitative analysis covers parameters such as mitochondrial length, mean square displacement (frequency and speed), and ultrastructural membrane fusion processes are highlighted. Additionally, we will dynamically illustrate other mitochondrial fluctuation phenomena, including tubular lasso formation, fission, and swelling. These findings collectively enable us to investigate and characterize the physiological state of cells throughout the photo-induced oxidative process, enabling comparisons between cells exposed to photo-induced oxidative stress and control cells.

## 2 Materials and methods

### 2.1 Cell models and culture

For all experiments, we employed HeLa cells. These cells were cultured in DMEM-Glutamax medium, supplemented with 10% calf fetal serum and 1% antibiotics (penicillin/streptomycin, all from Gibco), and kept at a temperature of 37°C in a 5% CO2 atmosphere. The cells were seeded in 35 mm Ibidi Petri dishes 2 days prior to conducting Fast-SIM experiments.

### 2.2 Cell oxidation

#### 2.2.1 Photosensitizer

Cellular lipid oxidation can be initiated through photoinduction using photosensitizing molecules such as Chlorin-e6 (Aubertin et al., 2013; Nadova et al., 2016; Zhu et al., 2021). Chlorin-e6 (Ce6) was obtained from Porphyrin Products (Logan, UT, USA). Stock solutions of the photosensitizer (1 mM) were prepared in 20 mM Na2HPO4 to ensure good solubility. The solution was handled in complete darkness.

#### 2.2.2 Cell loading

In preparation for Fast-SIM experiments, before the mitochondrial staining (as described above), cells were initially incubated with 0.5 µM of Ce6 for 30 min. Subsequently, they were rinsed with PBS and transferred to Gibco™ FluoroBrite™ DMEM medium.

#### 2.2.3 Irradiation

During the imaging experiment, cells were exposed to light under the microscope, utilizing a 488 nm wavelength that excites both the mitochondrial probe and Ce6. Since the Ce6-photosensitizers emission peaks at 665 nm, its fluorescence was not collected by our camera and did not interfere with the dye imaging.

### 2.3 Cell imaging

#### 2.3.1 Mitochondrial staining

For mitochondrial fluorescent staining, we used Mitotracker Green™ (from Molecular Probes). Cells, grown in Ibidi Petri dishes, were initially incubated with 200 nM of the dye at 37°C in a 5% CO2 environment for 30 min. Subsequently, they were washed two times with PBS before being suspended in FluoroBrite™ DMEM for measurement purposes.

#### 2.3.2 Fast-SIM imaging set-up and reconstruction

**FIGURE 1:**
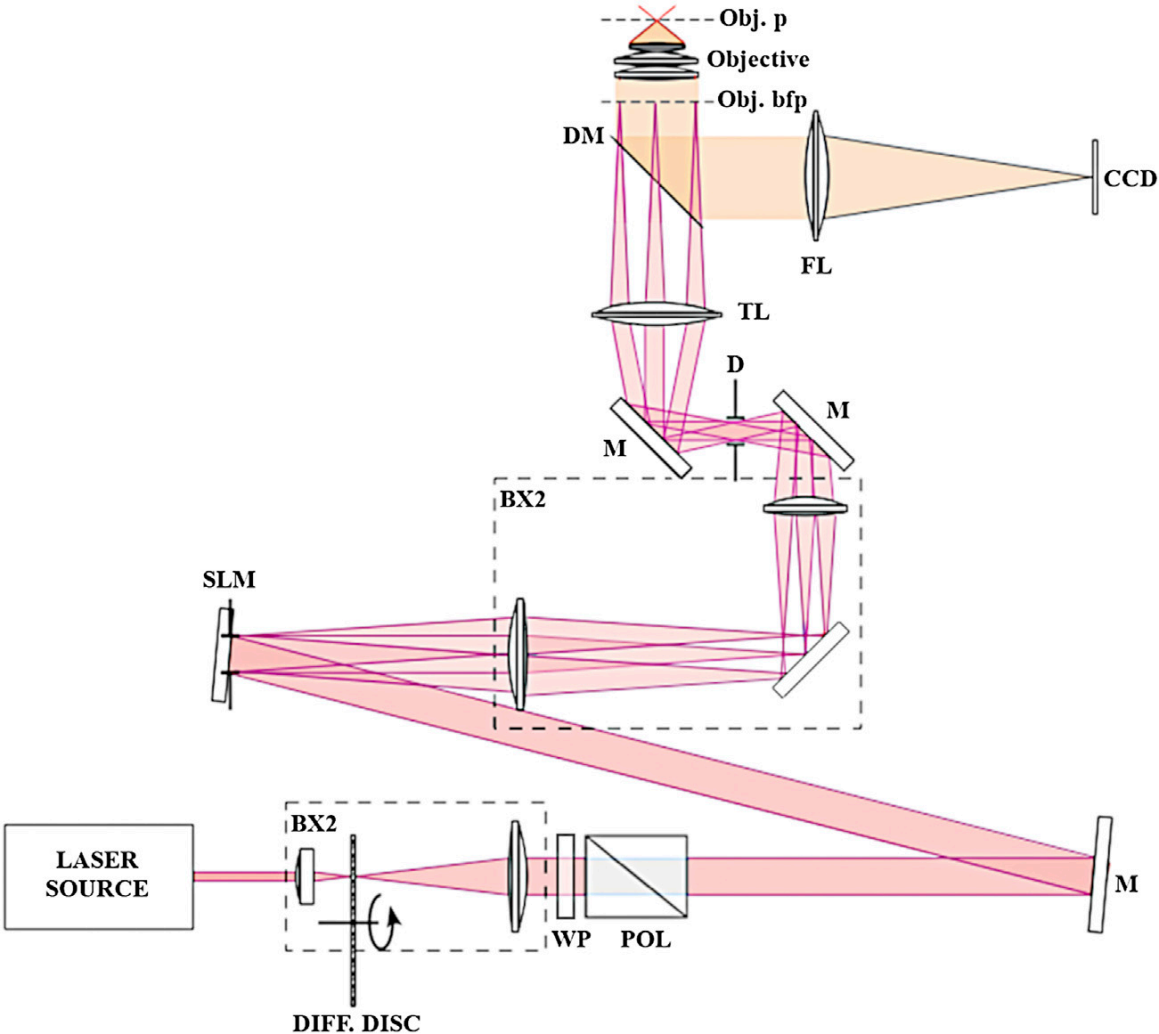
SIM Setup. Schematic representation of our SIM setup. LASER SOURCE, continuous-wave laser at 488 nm (Coherent); BX1, BX2, optical telescopes; DIFF. DISC, rotating diffusive semi-transparent disc for speckle pattern elimination in the recorded image; WP, half-wave plate for fine tuning of laser power at the sample; POL, Glan-Taylor prism polarizer, M, mirrors; SLM, spatial light modulator (HoloEye); D, diaphragm for higher-order diffracted beams suppression; TL, microscope tube lens; DM, dichroic mirror coupled with long-pass rejection filter; FL, focusing lens; Obj. bfp, objective lens back focal plane; Objective, ×60 NA 1.2 water immersion objective lens; Obj. p, objective lens focal plane; CCD, charge-coupled device camera (Andor Ixon 885).

Our SIM is a custom-made set-up (see Figure 1) based on a commercial epi-fluorescence microscope from Olympus (IX81). Fringes at the image plane are obtained by diffracting a CW 488 nm laser (Coherent) on a spatial light modulator (HoloEye), selecting only the zero order and two diffracted orders (+1,-1) in using a diaphragm, and focusing them in the objective back focal plane. Live cells images are acquired with a 60x NA1.2 water immersion objective. A periscope is allowing aligning the beams with the objective optical axis. We have chosen to use an SLM to avoid any mechanical displacement and ensure versatility for the pattern design (especially for its spatial frequency and orientation) and high raw data acquisition rate (maximum refresh rate of 60 Hz). Our SIM reconstruction algorithm is based on the assumption that out of focus fluorescence is not modulated by the illumination pattern. This requires confining the fringes within the depth of field of the microscope. This was achieved by reducing the spatial coherence of the laser source with a rotating diffuser. Images were acquired with an Andor Ixon 885 camera.

The mathematical foundation and the reconstruction algorithm, based on a myopic inversion problem, have already been described in previous articles (Orieux et al., 2012; Orieux et al., 2017). In the context of SIM algorithms, the process requires disentangling sub-spectra and ensuring adequate redundancy for accurate sub-spectra replacement typically, and necessitates a substantial number of input images. In contrast to conventional methods, our approach operates without the need for information redundancy. Four images contain enough information to effectively double the lateral spatial resolution and provide a combined estimation encompassing the super-resolved image, modulation parameters, and out-of-focus background. Thus, to ensure isotropic resolution enhancement with our technique, we acquired three images with a sinusoidal fringe pattern at three distinct orientations and one additional image with uniform illumination, i.e., a total of four raw images to reconstruct an enhanced resolution image, instead of 9 or 15 in classical SIM. This fast super resolution microscopy technique is particularly adapted to dynamic studies in living cells.

### 2.4 Data processing

**FIGURE 2:**
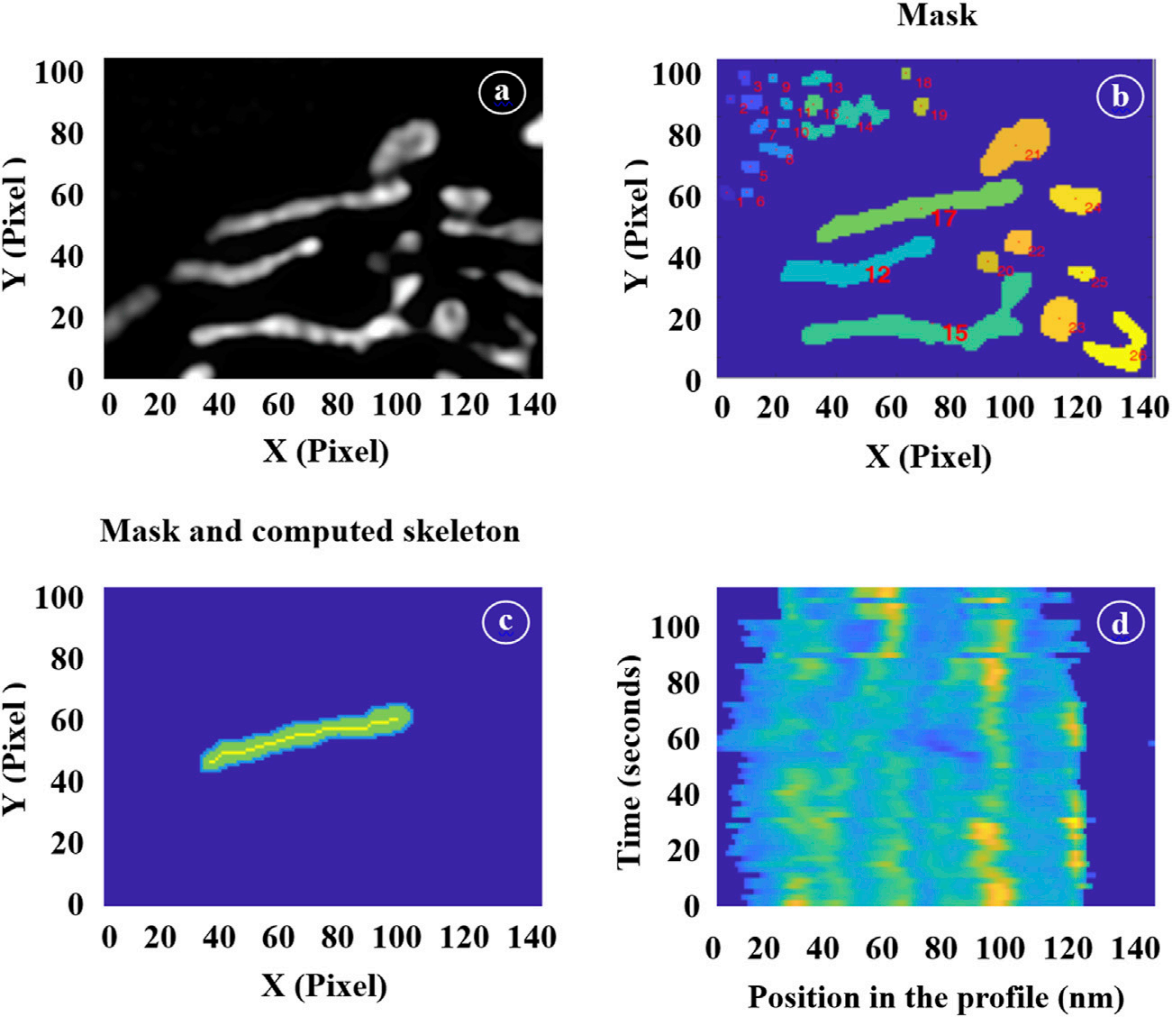
Illustration of the different image processing steps. **(A)** Selection of a region of interest in a wide-field image. **(B)** Mask generation. **(C)** Selection of individual mitochondrion masks and computation of their skeletons. **(D)** Time-lapse showing the positions of the inner mitochondrial structures.

The processing of image stacks was carried out sequentially (please, refer to Figure 2). First, a global mask was generated from the initial wide-field image using a standard Otsu threshold algorithm. A label was assigned to each mitochondrion within this mask, and a subset of them was manually selected for further processing. This selection depended on the overall image quality of the mitochondria throughout the entire movie and the ease of distinguishing them from neighboring structures. This step is illustrated in Figure 2A, which displays a portion of a full 1024 × 1024 pixel mask obtained from a wide-field image and 2b: each identified object is assigned a label, and individual masks were chosen as suitable candidates for subsequent processing. In our illustration, masks 1, 15, and 17, for instance, were chosen.

For each image within the SIM stack, we then extracted the skeleton of the selected mitochondria masks using a thinning algorithm. Figure 2C illustrates this step by displaying the individual mask labeled as 17 alongside its corresponding skeleton. Additionally, we applied morphological operations to these masks to extract various geometric parameters such as lengths, widths, and surface areas. The skeletons of the masks, when multiplied by the actual SIM image, provided profiles that were utilized to count the number of cristae and determine their positions using a multi-peak detection algorithm.

To ensure continuity from one image to the next, it was essential to track the same mitochondria. Therefore, at each step (n+1), we employed the individual masks generated in the preceding step (n). These masks were expanded by typically 5 pixels, serving as a buffer zone, and we applied the threshold to the n+1 image exclusively within these enlarged regions. This dilation strategy enabled us to track the movement of mitochondria as they transition from one frame to the next. The specific amount of dilation was determined empirically by closely observing the mitochondrial movement between consecutive frames. If the dilation was too conservative, there was a risk of omitting sections of rapidly moving mitochondria, while overly generous dilation might have incorporate parts of neighboring mitochondria into our individual masks. Strike the right balance was crucial for accurate tracking.

Since our interest extended to the movement of cristae within mitochondria, it became necessary to isolate this movement from the overall mitochondrial motion. For each individual mitochondrion, we have established a reference point using the position of the first skeleton profile. Subsequently, we have estimate the position of each subsequent profile by optimizing the correlation between these two profiles. This iterative process was applied to every successive profile. Figure 2D presents a vertical stack of profiles, with the initial one at the bottom of the figure. By identifying the local maxima in each profile, we have gained the ability to track the motion and relative positioning of the cristae. This approach allowed us to calculate their velocity and the frequencies of their orientation changes.

## 3 Results

### 3.1 Time-lapse Fast-SIM imaging of the cells

We employed Fast-SIM imaging to capture the dynamic behavior of the intracellular mitochondrial network and of the ultrastructure of the inner mitochondrial membrane. Our imaging sessions consisted of movies ranging from 30 to 60 frames, with each frame being acquired in just 4 × 100 milliseconds. A brief pause of 1–2 s separated each consecutive frame. We conducted Fast-SIM imaging for both control cells and cells submitted to photo-induced oxidation (please refer to supplementary data for detailed video).

#### 3.1.1 Capturing of the dynamic behavior of the mitochondrial inner membrane

**FIGURE 3:**
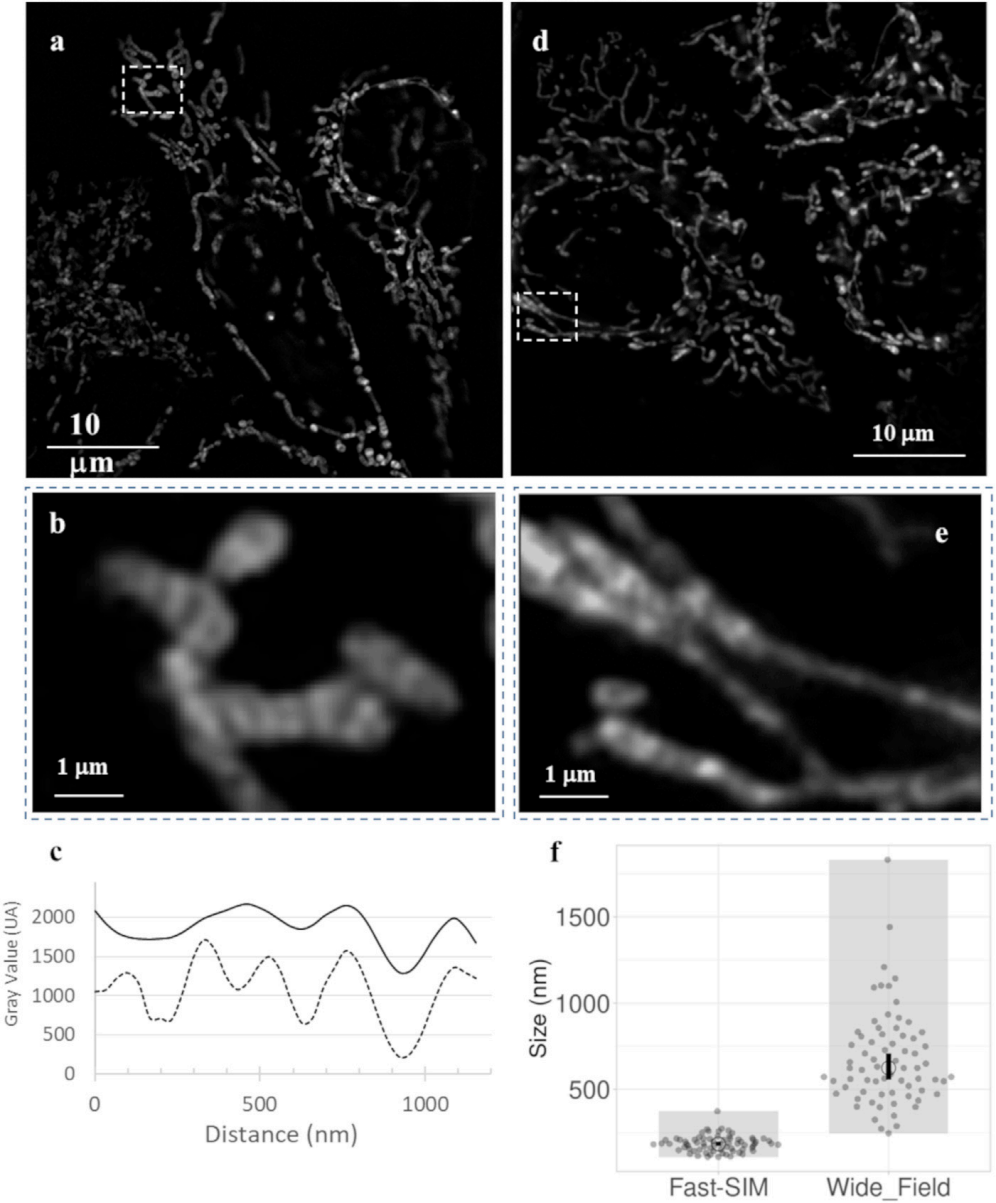
Fast-SIM images of mitochondria in HeLa Cells (Full Time-Lapse sequences in Supplementary Data). **(A)** Typical frame from a movie of control cells, **(B)** zoomed view of a selected area, and **(C)** plots of fluorescence intensity profiles obtained with Fast-SIM (dashed line) and wide-field imaging (solid line) for the selected mitochondria. **(D)** Typical frame from a movie of cells under photo-induced oxidative stress, and **(E)** zoomed views of selected areas. **(F)** Distribution of measured peak widths (in nm) on intensity profiles of in 140 mitochondria demonstrating a significant improvement in resolution (*p*-value equals 0.0001).

Figure 3 displays typical image frames for reference. It is important to highlight that the wide-field illumination feature of SIM allows us to simultaneously observe multiple individual cells, usually ranging from 3 to 6 cells, all with improved optical resolution. This capability opens the door to conducting statistical analyses of mitochondrial membrane dynamics under precisely controlled conditions. Figures 3B, E provide zoomed-in views of the insets (dashed-line rectangles) depicted in Figures 3A, B, respectively. In these images, we can clearly discern mitochondrial sub-structures. Consistent with the maximal theoretical spatial resolution of our SIM setup, these inner mitochondrial membrane structures exhibit a width of approximately 150 nm, with an interspace of roughly similar, as indicated by intensity profiles derived from Fast-SIM imaging of a single mitochondrion. In contrast, the wide-field resolution is approximately 400 nm (Figure 3C).

#### 3.1.2 Effective resolution gain

To evaluate the actual improvement in resolution under our experimental conditions, we traced intensity profiles along the mitochondrial skeletons. In Figure 3F, the sizes presented represent the Full Width at Half Maximum (FWHM), calculated based on the local Gaussian-like intensity variations. In the wide-field images, the profiles lack clearly defined bands, resulting in highly variable values. These profiles have low amplitude, with a mean FWHM value of 675 nm and a standard deviation of 275 nm, indicating the absence of clearly distinguishable distinct structures within the mitochondria. In contrast, the Fast-SIM images exhibit distinct peaks that characterize the inner membrane structure. We estimated the mean size of these structures in Fast-SIM images to be 184 ± 45 nm. This analysis underscores the substantial enhancement in spatial resolution achieved with Fast-SIM imaging.

Comparing these substructures with images obtained *via* transmission electron microscopy (TEM) or STED nanoscopy of mitochondria within similar HeLa cells (Große et al., 2016; Stephan et al., 2019), it appears that these structures do not likely correspond to the visualization of individual cristae but instead seem to represent distinct groups of cristae, as has also been suggested by the authors of some of these previous works (Große et al., 2016). Nevertheless, the use of very low irradiation power (0.3 W/cm2) and the higher-frequency acquisition rate of Fast-SIM (up to 2 frames per second, with each frame captured in 4 × 100 milliseconds) enables extended and non-invasive dynamic studies of these resolved intramitochondrial cristae-related structures.

#### 3.1.3 Non-invasiveness and oxidation control

To validate the non-invasive nature of our method on cells, we examined Fast-SIM movies portraying the mitochondrial network in both control cells and photo-induced cells, revealing their remarkable dynamism. It is noteworthy that the mitochondrial shape and dynamics in control cells remain unchanged even after 60 s of irradiation. In contrast, photo-sensitized cells subjected to irradiation exhibit conspicuous alterations in mitochondrial shape and dynamics (please see supplementary movies for visual reference). After approximately 60 s of illumination, distinctive indicators of oxidation, such as mitochondrial swelling, become discernible in certain mitochondria. This oxidative process persists until its culmination, as evidenced by its impact on the entire mitochondrial network upon completion of the measurements (after 120 s). At various levels, these types of intracellular stress responses of mitochondria are distinguishable in many images obtained by other super-resolution approaches.

### 3.2 Statistical analysis

#### 3.2.1 Mitochondrial shape changes

**FIGURE 4:**
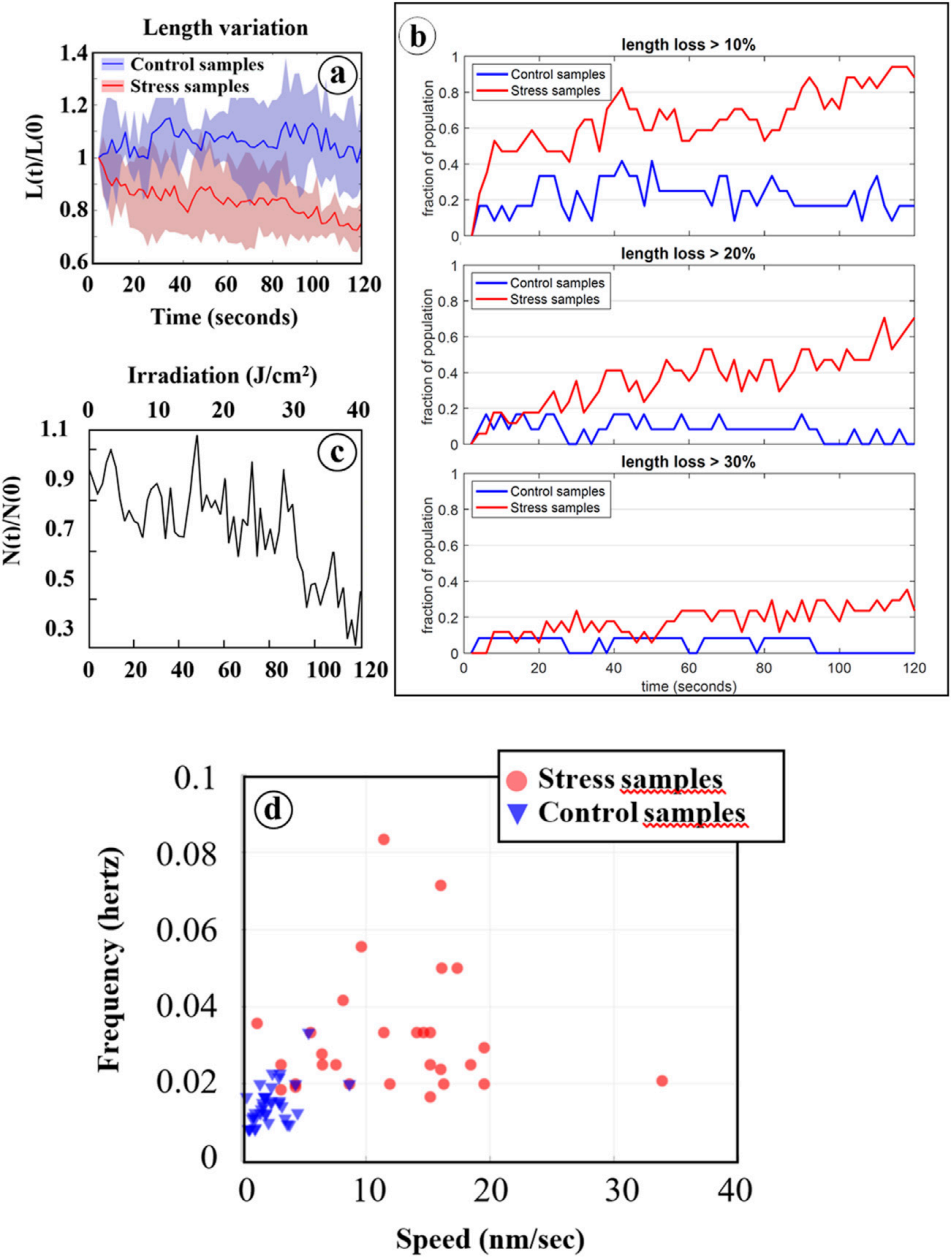
Statistical analysis based on data processing of Fast-SIM movies. **(A)** Average relative length of mitochondria (L[t]/L[0]) as a function of irradiation time are reported for both control cells (blue line) and cells pre-incubated with Ce6 (red line). The blue and red areas represent the distribution of all individual values. **(B)** Evolution of the fraction of mitochondria that have lost at least 10%, 20%, and 30% of their length during irradiation, for both control cells (in blue) and cells with Ce6 (in red). **(C)** Mitochondrial cristae fusions under photo-induced oxidative stress: Evolution of the relative number of “cristae” (N[t]/N[0]) for mitochondria that have lost at least 30% of their length after 120 s of irradiation (see Figure 4B. **(D)** Two-dimensional representation of mitochondrial cristae fluctuations as a function of the frequency of orientation changes in their motion and their motion speeds.

Various structural parameters were quantitatively analyzed based on data extracted from Fast-SIM movies, as outlined in the materials and methods section. Initially, we examined the evolution of mitochondrial length during irradiation, and the results are presented in Figures 4A, B. While the cellular response is not uniform, with some mitochondria reacting more swiftly and intensely than others, a statistically significant mitochondrial shortening was measured in cells exposed to oxidative stress, in contrast to control cells. Furthermore, both the number of affected mitochondria and the rate of this process increased with the degree of oxidation (i.e., with irradiation time). In cells submitted to photo-induced oxidation, approximately 60% of the mitochondria had lost at least 20% of their length after 120 s of irradiation. This shortening, previously described as a hallmark of cellular oxidative stress, serves as a characteristic marker of oxidation level (Zhu et al., 2021). Therefore, the absence of such an effect on control cells, even after prolonged irradiation, provides compelling evidence for the non-invasive nature of our Fast-SIM approach.

To assess the impact of irradiation on the affected mitochondria, the total mitochondrial population was categorized into three groups based on the extent of length reduction: mitochondria with less than a 20% reduction were classified as poorly affected (population 0), those with a 20%–30% length reduction were considered moderately affected (population 1), and those with more than a 30% reduction were deemed strongly affected (population 2).

#### 3.2.2 Dynamics within oxidized mitochondria

Subsequently, we conducted an analysis of the dynamics of inner membrane structures, revealing a distinctive reduction in the number of these structures, corresponding to the fusion of adjacent structures during the photo-induced mitochondrial shortening process. These fusion events became particularly evident with prolonged irradiation (t > 80 s) in mitochondria belonging to population 2 (Figure 4C). The mechanisms underlying these events remain unidentified, but intriguingly, they are preceded by a period of increased fluctuations in the inner mitochondrial structures when compared to control cells. These fluctuations are characterized by elevated speed and frequency of orientation modifications of the motion in response to photo-induced oxidative stress (Figure 4D). Specifically, these motions exhibited speeds of less than 10 nm per second with frequencies below 0.02 Hz in control cells, whereas photo-sensitized cells displayed speeds ranging from 10 to 20 nm per second (and even higher occasionally) with frequencies ranging from 0.02 up to 0.1 Hz. These dynamic changes are thus closely associated with irradiation and should be considered as early consequences of the photo-induced oxidation process.

## 4 Discussion

**FIGURE 5:**
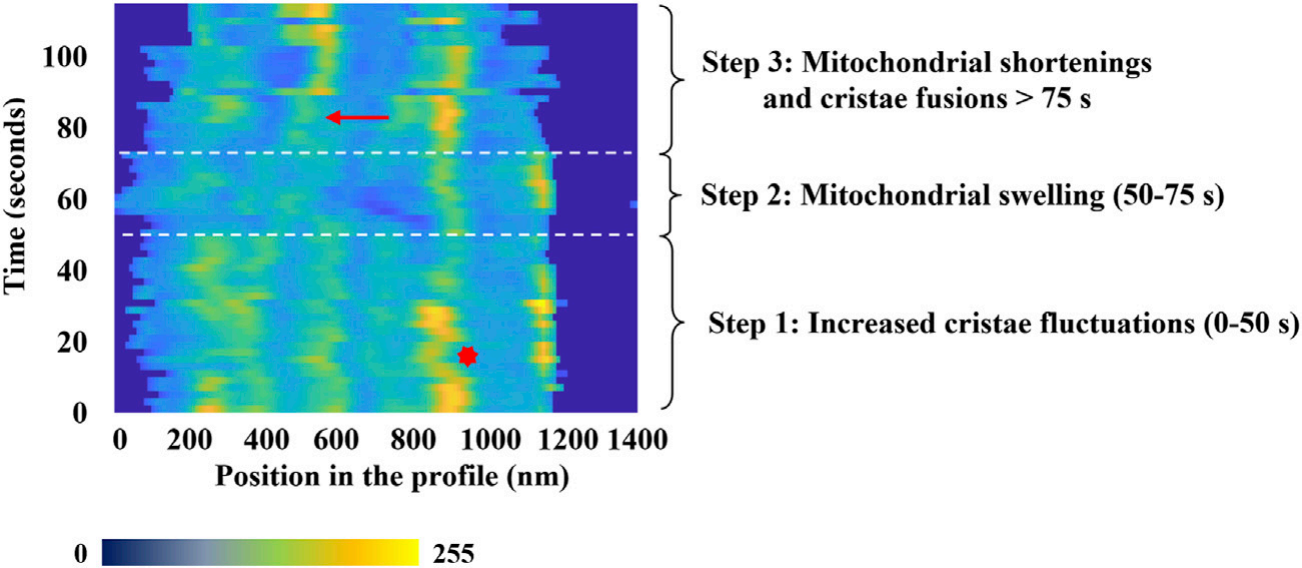
Characteristic temporal evolution of the inner fluorescence intensity profile from a selected mitochondrion. Time-lapse of the different phases of mitochondrial dynamics highlights the mitochondrial membrane remodeling process under oxidative Stress. On *x*-axis, the fluorescence intensity is represented using a false color scale, where dark blue corresponds to 0 and yellow represents 255. The red arrow indicates a cristae-related fusion event, and the red asterisk a typical change of the cristae structures motion.

Considered as a whole, our observations and measurements offer a coherent and comprehensive scenario that delineates the events occurring within mitochondria exposed to a high level of oxidative stress. The dynamics of mitochondrial membrane remodeling under oxidative stress can be summarized in three distinct phases, as illustrated in Figure 5.

**FIGURE 6:**
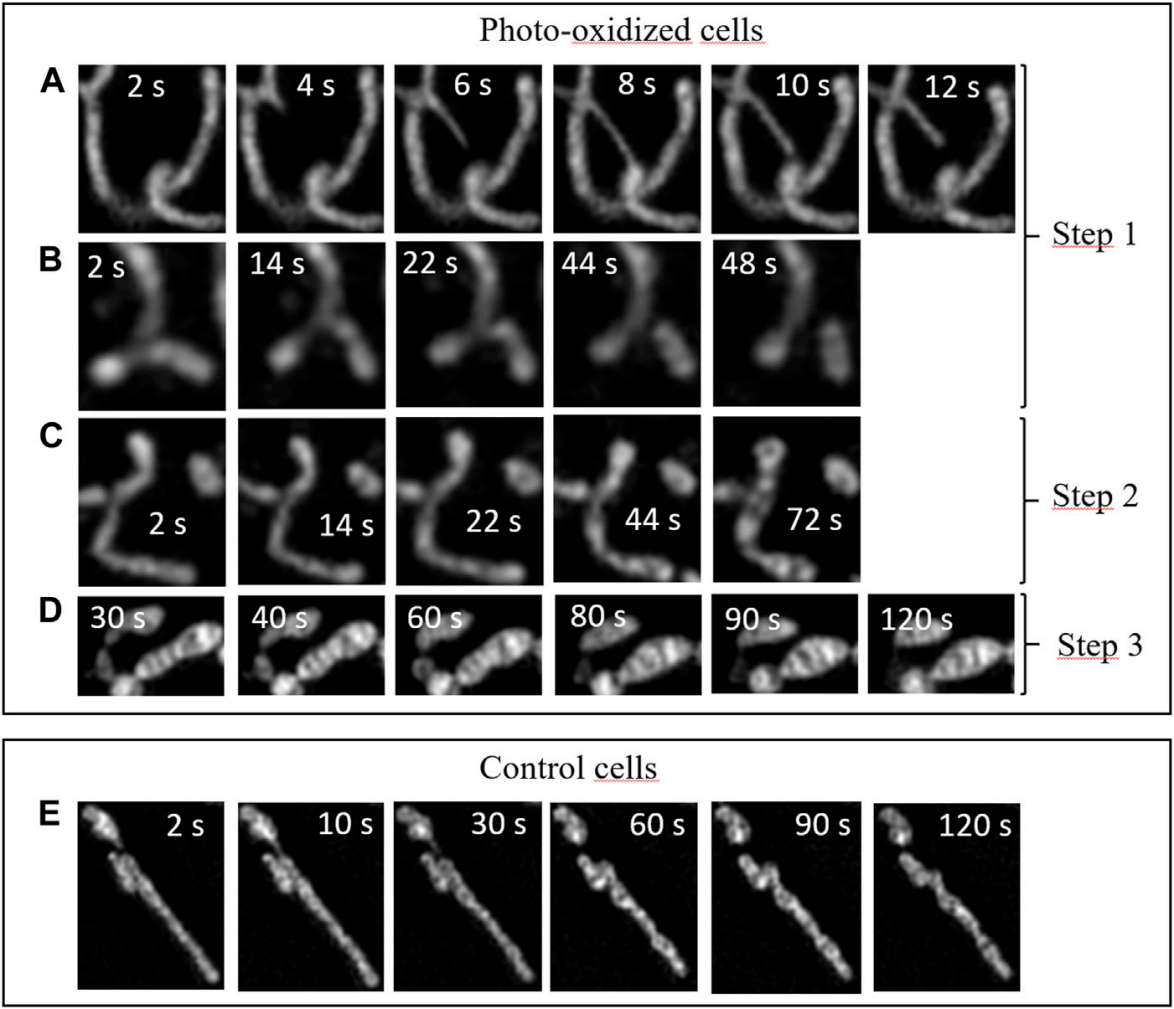
Dynamics of intracellular fluctuations in the mitochondrial network in HeLa Cells under oxidative stress and for control cells. Time-lapse sequences correspond to specific zoomed areas of Fast-SIM movies. Insert: Time scale in seconds. **(A)** Tubular lasso protrusions, **(B)** fission events, **(C)** swelling, **(D)** fusion of inner cristae structures, **(E)** control cells.

• **Initial Phase (≈0 to 50 s):** This phase is marked by increased fluctuations in the inner mitochondrial membrane structures, characterized by rapid motion and frequent orientation changes around a fixed average position (refer to the red asterisk (*) in Figure 5). Preliminary destabilizations of these structures, including internal structure fission (see Figure 6B), can be observed toward the end of this phase. However, a fundamental question remains unresolved: do these increased fluctuations solely reflect membrane alterations under oxidative stress, or might they be related to an enhanced antioxidant activity of the mitochondrial respiratory chain, attempting to counterbalance the effects of photo-induced reactive oxygen species (ROS) production?
• **Swelling Phase (≈50 to 75 s):** The second phase is characterized by a global swelling of the mitochondria. The decrease in image contrast is interpreted as a consequence of both the transient loss of the focus plane due to the swelling process and the destabilization of internal structures.
• **Final Phase (after 75 s):** During this phase, inner structure fusions occur during the shortening of mitochondrial length. Fluorescence intensity and focus plane are recovered, and fusion of internal structures (indicated by the red arrows (→) in Figure 5) can be observed, which implies a decrease in their number.

The enhanced spatiotemporal resolution provided by our approach allowed us to capture distinct events characteristic of each of these phases in our video sequences (Figure 6). Consequently, we were able to delve deeper into the mechanisms underlying the phenomena outlined in this scenario.

During the initial phase, numerous mitochondria extend elongated tubular lasso-shaped structures, each with diameters ranging from ∼100 to 150 nm (Figure 6A). These tubular structures, referred to as mitochondrial nanotunnels, have previously been identified as conduits for inter-mitochondrial communication, facilitating molecular exchanges among mitochondria. We measured the timescales of these lasso protrusions, which are still a subject of discussion, and found that they typically last for just a few seconds before undergoing retraction. Their size may facilitate a selective size-based diffusion of biomolecules along these nanotunnels, in accordance with emerging evidence suggesting that such communication may emanate from mitochondria with impaired respiratory chains, seeking assistance in maintaining their functional activity (Vincent et al., 2017).

During this initial phase, we also observed Mitochondrial Fission events (Figure 6B). This process unfolds over a timescale of tens of seconds, as depicted in the Figure. It is known to function as a mechanism for mitochondrial proliferation within cells and is associated with the regulation of mitochondrial membrane tension (Mahecic et al., 2021). During oxidative stress, these events can be seen as mechanisms through which cells strive to boost their metabolism and respond to the mechanical stress induced by the photo-oxidation of the membrane (Heuvingh and Bonneau, 2009; Bour et al., 2019), both in an effort to restore a stable physiological state.

The second phase corresponds to a higher level of cellular oxidation, during which mitochondria initiate a profound reorganization. As illustrated in the sequence shown in Figure 6C, mitochondrial swelling occurs prior to the appearance of 'cristae fusion’ events. This swelling is a hallmark of cells that are heavily damaged and engaged in a cellular pathway ultimately leading to irreversible apoptotic or necrotic cell death (Peng and Jou, 2004). In accordance with our scenario, this step occurs just before the final phase.

Subsequently, the third phase encompasses drastic alterations, including mitochondrial shortening and an increase in their width, as previously detailed and visible in Figure 6D (Miyazono et al., 2018). These distinct modifications coincide with a reduction in the number of cristae-related inner structures, throughout the oxidative process. Importantly, the reduction in the number of mitochondrial cristae due to cellular oxidative stress has been observed previously using cryo-TEM (Pamenter et al., 2013). Our findings provide fresh insights into the dynamics of these events: during the initial 60–80 s of irradiation, mitochondrial structures remain relatively stable, only to undergo sudden and dramatic transformations at both the organelle scale (length, width) and the sub-mitochondrial level. This suggests that under oxidative stress, the mitochondrial network would initially attempt to adapt its activity to counteract the excessive production of ROS. However, beyond a certain threshold, rapid and drastic changes in mitochondrial structures occur, exhibiting characteristic features associated with apoptotic or necrotic pathways. For comparison, Figure 6E provides an illustration of the typical dynamics of mitochondria in control cells. None of the sub-mentioned specific events are observed (refer to the supplementary data for a detailed video of whole control cells).

In conclusion, our study provides compelling evidence that Fast-SIM stands out as a method of choice for non-invasive, long-term, and dynamic investigations of sub-mitochondrial structures across a broad field of observation. This approach has empowered us to track dynamic alterations in mitochondrial ultrastructures, conduct statistical analyses of the observed cellular changes induced under controlled oxidative stress, and quantify these transformations. We have delineated three consecutive phases: an initial phase marked by heightened dynamics within the mitochondrial network, a subsequent phase characterized by mitochondrial swelling, which may be linked to the initiation of apoptotic or necrotic pathways, and a final phase featuring profound alterations in shapes of mitochondria and of their inner membrane structures.

These findings are consistent with previous research conducted using other enhanced-resolution microscopy techniques (Peng and Jou, 2004; Vincent et al., 2017; Miyazono et al., 2018; Mahecic et al., 2021; Zou et al., 2021). Nevertheless, to the best of our knowledge, none of these methods are suitable for the comprehensive monitoring of dynamic changes in mitochondrial membrane structures within living cells, offering both the necessary temporal and spatial resolutions while avoiding uncontrolled artifacts. We were able to underscore the remarkable dynamics exhibited by stressed mitochondria, which correspond to substantial remodeling at both the organelle and nanoscale levels. This study opens up new avenues of exploration in the realm of mitochondrial disorders, which play a pivotal role in numerous pathological conditions, with a particular emphasis on unraveling the intricate interplay between structural modifications and physiological changes. Furthermore, our Fast-SIM method, as other SIM approaches, is versatile and can be used in the study of other intracellular organelle structures. For example, the nanoscale distribution patterns of subcellular structures in platelets, including granules and microtubules, were successfully imaged using structured illumination super-resolution fluorescence microscopy (Xu et al., 2023).

## Data Availability

The original contributions presented in the study are included in the article/supplementary material, further inquiries can be directed to the corresponding author.
