# Supplementary material for: Dynamics of mitochondrial membranes under photo-oxidative stress with high spatiotemporal resolution
Source: Front Cell Dev Biol. 2023 Nov 17;11:1307502. doi: 10.3389/fcell.2023.1307502 (PMC10691360; doi:10.3389/fcell.2023.1307502)
Supplement: Supplementary file 2 [file Table1.DOCX]

Supplementary data

Dynamics of mitochondrial membranes under photo-oxidative stress with high spatiotemporal resolution

The supplementary data corresponds to two Fast-SIM movies of mitochondria in HeLa Cells. The cells were stained with Mitotracker GreenTM (from Molecular Probes). Cells, grown in Ibidi Petri dishes, were initially incubated with 200 nM of the dye at 37°C in a 5% CO2 environment for 30 minutes. Subsequently, they underwent two PBS washes before being suspended in FluoroBrite™ DMEM for imaging with our fast-SIM set-up (x60 NA 1.2). The excitation wavelength was 488 nm, and the reconstructed 32 bits-images were 1040x1040 pixels.

SuppMovie1.avi corresponds to control HeLa Cells.

SuppMovie2.avi corresponds to HeLa Cells under photo-oxidative stress induced by the Ce6-photosensitizer (the cells were incubated with 0.5 µM of Ce6 for 30 minutes prior to the MitoTracker staining).
